# Nck-associated protein 1 associates with HSP90 to drive metastasis in human non-small-cell lung cancer

**DOI:** 10.1186/s13046-019-1124-0

**Published:** 2019-03-11

**Authors:** Yuanping Xiong, Leilei He, Chloe Shay, Liwei Lang, Jenni Loveless, Jieqing Yu, Ron Chemmalakuzhy, Hongqun Jiang, Manran Liu, Yong Teng

**Affiliations:** 1 Department of Otolaryngology Head and Neck Surgery, First Affiliated Hospital of Nanchang University, Nanchang, Jiangxi China; 2 Department of Oral Biology and Diagnostic Sciences, Dental College of Georgia, Augusta University, 1120 15th Street, Augusta, GA 30912 USA; 3 Division of Endocrinology and Diabetes, Department of Pediatrics, School of Medicine, Emory University, Atlanta, GA USA; 4 Department of Biology, College of Science and Mathematics, Augusta University, Augusta, GA USA; 5 Key Laboratory of Laboratory Medical Diagnostics Designed by Chinese Ministry of Education, Chongqing Medical University, Chongqing, China; 6 Georgia Cancer Center, Department of Biochemistry and Molecular Biology, Medical College of Georgia, Augusta University, 1120 15th Street, Augusta, GA 30912 USA; 7 Department of Medical Laboratory, Imaging and Radiologic Sciences, College of Allied Health, Augusta University, 1120 15th Street, Augusta, GA 30912 USA

**Keywords:** NCKAP1/NAP1, HSP90, lung cancer, metastasis, protein stability

## Abstract

**Background:**

Metastatic lung cancer is a life-threatening condition that develops when cancer in another area of the body metastasizes, or spreads, to the lung. Despite advances in our understanding of primary lung oncogenesis, the biological basis driving the progression from primary to metastatic lung cancer remains poorly characterized.

**Methods:**

Genetic knockdown of the particular genes in cancer cells were achieved by lentiviral-mediated interference. Invasion potential was determined by Matrigel and three-dimensional invasion. The secretion of matrix metalloproteinase 2 (MMP2) and MMP9 were measured by ELISA. Protein levels were assessed by Western blotting and immunohistochemistry. Protein-protein interactions were determined by immunoprecipitation. An experimental mouse model was generated to investigate the gene regulation in tumor growth and metastasis.

**Results:**

Nck-associated protein 1 (NAP1/NCKAP1) is highly expressed in primary non-small-cell lung cancer (NSCLC) when compared with adjacent normal lung tissues, and its expression levels are strongly associated with the histologic tumor grade, metastasis and poor survival rate of NSCLC patients. Overexpression of NAP1 in lowly invasive NSCLC cells enhances MMP9 secretion and invasion potential, whereas NAP1 silencing in highly invasive NSCLC cells produces opposing effects in comparison. Mechanistic studies further reveal that the binding of NAP1 to the cellular chaperone heat shock protein 90 (HSP90) is required for its protein stabilization, and NAP1 plays an essential role in HSP90-mediated invasion and metastasis by provoking MMP9 activation and the epithelial-to-mesenchymal transition in NSCLC cells.

**Conclusions:**

Our insights demonstrate the importance and functional regulation of the HSP90-NAP1 protein complex in cancer metastatic signaling, which spur new avenues to target this interaction as a novel approach to block NSCLC metastasis.

**Electronic supplementary material:**

The online version of this article (10.1186/s13046-019-1124-0) contains supplementary material, which is available to authorized users.

## Background

Non-small-cell lung cancer (NSCLC) accounts for 85%-90% of all lung cancer cases, and is the most common cause of cancer-related deaths worldwide [1]. With modern cancer therapies, the 5-year survival rate for all lung cancer stages is around 16%. Metastatic NSCLC is an incurable disease and the most common sites of metastases are the contralateral lung (40-50%), liver (20%), adrenal gland, bones and brain [2, 3]. High mortalities of NSCLC are the result of metastasis; therefore, reducing metastasis is the key to curtailing the rate of death from NSCLC. However, not much is known about specific molecular mechanisms underpinning NSCLC metastasis.

Nck-associated protein 1 (NAP1/NCKAP1), a protein that associates with the Src homology 3 (SH3) domain of NCK protein both *in vitro* and in intact cells, was found to localize along the lamellipodia and to mediate contact-dependent cell migration [4]. In the cell, NAP1 together with four components: CYFIP1, ABI2 (or the orthologs ABI1 and ABI3), HSPC300/BRICK1 and WASF1/SCAR (or the orthologs WASF2 and WASF3), form the WASF regulatory complex (WRC) [5, 6]. Following stimulation with cytokines or growth factors, this 5-subunit protein complex is recruited to the membrane and triggered to release its inhibitory effects on WASF proteins, which are involved in the formation of the actin cytoskeleton through interaction with the Arp2/3 complex [7, 8]. Increasing evidence has shown that NAP1 is critical for cell motility and adhesion by driving actin assembly and polymerization and lamellipodia formation [5, 9, 10], which are associated with the development of invasion and metastasis phenotypes. Particularly in breast cancer, univariate analysis reveals that high expression of NAP1 is strongly correlated with poor metastasis-free survival of patients with breast cancer, suggesting NAP1 as an independent prognosis factor [11]. WASF3 is a tumor metastasis driver in breast cancer, and its knockdown leads to a significant reduction in metastatic breast cancer cell invasion and metastasis in mice [5]. Our previous studies further demonstrated that NAP1 is required for the protein stability of WASF3 in breast cancer cells, implicating that NAP1 is a critical regulator in favor of breast cancer metastasis [5].

Although the function of NAP1 is associated with the invasive potentials of cancers and therefore their aggressive nature, there is lack of preclinical evidence and mechanisms reporting the importance of NAP1 during the metastasis and progression of NSCLC. Here, we reveal that NAP1 is sufficient to drive NSCLC invasion and metastasis and that this ability is associated with the function of the chaperone protein HSP90. HSP90 stabilizes the NAP1 protein by preventing it from ubiquitin-proteasome-dependent degradation. Additionally, NAP1 provoked activation of MMP9 and upregulation of Vimentin in NSCLC cells, which was required for HSP90-mediated metastasis. These findings reveal further insight into the mechanism of NAP1-mediated metastasis in NSCLS, which would be a potential therapeutic target to combat advanced lung cancer.

## Methods

### Human primary lung specimens and cell lines

NSCLC cell lines H460 and H661 were directly purchased from ATCC and were maintained in culture no more than 10 passages according to the supplier’s instructions. A paraffin-embedded lung carcinoma tissue array was obtained from US Biomax (Rockville, MD). Human primary lung tissue specimens of paraffin-embedded tissue blocks were obtained from the First Affiliated Hospital of Nanchang University, China. Specimens were collected and processed in compliance with protocols approved by the Institutional Review Board of Nanchang University. Human subjects provided informed consent in the course of this research.

### Reagents, DNA constructs and standard assays

Geldanamycin (GA), novobiocin, 17-allylamino-demethoxygeldamycin (17-AAG), cycloheximide (CHX) and MG132 were purchased from Sigma-Aldrich (St Louis, MO). The pLKO.1-puro TRC non-targeting control shRNA (shCONT) and shRNAs targeting NAP1 (shNAP1) or HSP90 (shHSP90) were obtained from Dharmacon Inc. (Lafayette, CO). ViraPower Lentiviral Packaging Mix contains an optimized mixture of the three packaging plasmids (pLP1, pLP2, and pLP/VSVG) was obtained from Invitrogen (Carlsbad, CA). The full-length Flag-tagged human NAP1 and HSP90 were cloned into pCDH-CMV-MCS-EF1-Puro (System Biosciences, Mountain View, CA) vector. Transient transfection, lentiviral infection and quantitative real-time RT-PCR (qRT-PCR) analysis, were carried out as previously described [5, 12]. Primer sequences for qRT-PCR assays were as follows: NAP1 forward primer, 5’-TCAAGAAGGCATGTGGAGACC-3’ and NAP1 reverse primer, 5’-CGGGTTTCTACAGCAGGGAA-3’; β-actin forward primer, 5’-TCCCTGGAGAAGAGCTACGA-3’ and β-actin reverse primer, 5’-AGCACTGTGTTGGCGTACAG-3’.

### Immunohistochemistry (IHC)

Tissue sections were deparaffinized with xylene and rehydrated with distilled water through a graded alcohol series. Tissue antigens were retrieved and the slides were subjected to IHC analysis for NAP1 expression using the ABC Elite Kit and the DAB Kit (Vector laboratories, Burlingame, CA) as previously described [13, 14]. The intensity of immunostaining was scored using the Image-Pro Plus software and presented as integrated optical density (IOD).

### Cycloheximide (CHX) chase assays and phalloidin staining

For CHX chase assays, cells expressing shCONT or shHSP90 were treated with 100 μg/ml of CHX for the indicated hours. Western blotting was then performed to determine the half-life of the NAP1 protein. For phalloidin staining, cells were fixed with 3.7% formaldehyde in PBS for 15 min and stained with Texas-red phalloidin (Molecular Probes, Eugene, OR) for 30 min, and then visualized using a Zeiss LSM 410 confocal microscope. In all quantifications, only those cells presenting with free borders were considered, and at least 100 cells from randomly selected fields were evaluated.

### Matrigel invasion and three-dimensional (3D) invasion assays

Matrigel invasion assays were performed by 8-μm pore size Transwells (BD biosciences, San Jose, CA) with pre-coated Matrigel as previously described [5, 12, 14]. Briefly, 5 × 10^4^ serum-starved cells were seeded in triplicate onto the upper chamber, and 10% FBS was added to the lower chamber. After 24 hours, non-invaded cells in the top insert were carefully removed with a cotton swab, and the chambers were fixed in 100% methanol and stained with 0.2% crystal violet. Membranes were excised from the inserts and mounted on microscope slides, and cells in 10 random fields were counted. For 3D invasion assays, 5×10^4^ HN12 cells diluted in 50% Matrigel (BD biosciences) were seeded in the center hole of SeedEZ™ 3D ring (Lena Bioscience, Atlanta, GA). After 10 days of culture, cells invaded in SeedEZ™ scaffold were stained with Texas-red phalloidin (Invitrogen) and imaged under a fluoresce microscope (Zeiss).

### Western blotting and immunoprecipitation (IP)

Western blotting was performed as we previously described [5, 12, 14]. Briefly, electrophoresis was performed on 8-12% SDS-PAGE gel and the proteins were transferred to nitrocellulose membrane. The membranes were incubated with the primary antibodies overnight at 4°C and with secondary antibodies for 1 hour at room temperature. To visualize peroxidase activity, ECL reagents from Bio-Rad (Hercules, CA) were used according to the manufacturer's instructions. For IP assays, the lysed cells were incubated with the indicated antibodies, followed by addition of protein A/G Sepharose® beadsbeads (Amersham Biosciences, South San Francisco, CA) overnight at 4 °C on a rotating platform. In these studies, primary antibodies that recognize NAP1, ubiquitin and β-Actin were purchased from Novus Biologicals (Littleton, CO). Antibodies against Vimentin and E-cadherin were ordered from Cell Signaling Technology (Beverly, MA). Antibodies that recognize HSP90 and FLAG were obtained from Enzo Life Sciences (Farmingdale, NY) and Sigma Aldrich (St Louis, MO), respectively.

### Active matrix metalloproteinase 2 and 9 (MMP2 and 9) detection

Cells were cultured in 12-well plates for 24 hours. The media was then collected and centrifuged for 10 min at 10,000g. The pro and active forms of MMP2 and MMP9 levels released into the media were measured using a Fluorokine MAP human MMP2 and MMP9 kit (R&D Systems, MN) according to the manufacturer's instructions. The fluorescent signal in the sample was determined using a BioRad analyzer at excitation/emission wavelengths of 340 nm/465 nm.

### Animal studies

Six-week-old NSG (NOD.Cg*-Prkdc*^*scid*^ *Il2rg*^*tm1Wjl*^*/SzJ*) mice were purchased from the Jackson Laboratory (Bar Harbor, ME), and all procedures were performed based on the AU institutional Animal Care and Use Committee (IACUC)-approved protocols. To generate a xenotransplantation model, 5×10^5^ H460 cells were suspended in 100 μl PBS and injected subcutaneously into the right flanks of mice. The tumor-bearing mice were sacrificed on treatment day 42, and the lungs were removed and processed for histopathological analysis with H&E staining.

### Bioinformatics and statistical analysis

NAP1 expression data in different cancer types were obtained from the website Oncomine (www.oncomine.org). To determine the influence of NAP1 expression on the overall survival of lung cancer patients, integrated available genome-level transcriptomic datasets from the Kaplan Meier-plotter were assessed [15]. All percentiles between the lower and upper quartiles of gene expression were computed for the Kaplan Meier method and the log-rank test, and the best performing threshold was used as a cut-off point for high and low groups of NAP1 gene expression. For other statistical analysis, data were analyzed using two-tailed Student’s *t*-test by comparing with the control group. For multiple comparisons, data were analyzed via analysis of variance (ANOVA) with the Tukey-Kramer Multiple Comparisons Test. Continuous normally distributed data are expressed as the means ± SDs and the differences of *p <* 0.05 was considered statistically significant.

## Results

### NAP1 expression levels are strongly associated with NSCLC metastasis

**Fig. 1:**
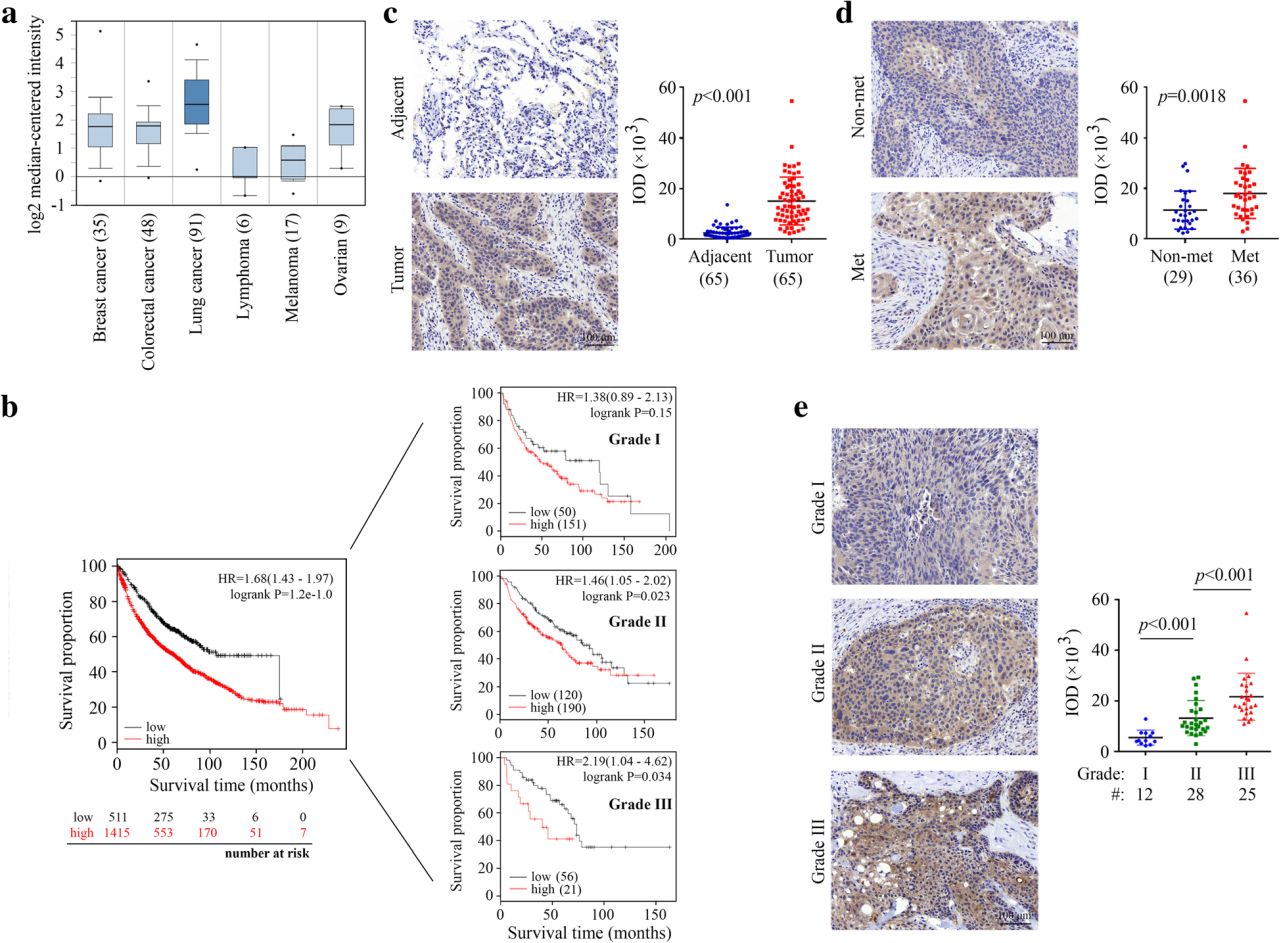
High expression levels of NAP1 correlate with low survival and poor prognosis in NSCLC patients. (**a**) Analysis of NAP1 expression in different types of cancer using Oncomine database. (**b**) Kaplan-Meier plot of overall survival shown for lung cancer patients with high (red) and low (black) expression levels of NAP1. (**c-e**) NAP1 expression levels determined by IHC with anti-NAP1 antibody in human primary NSCLC and paired adjacent normal lung tissues (**c**), in non-metastatic (Non-met) and metastatic (Met) NSCLC (**d**), and in primary NSCLC with different tumor grades (**e**). In (**c-e**), representative IHC results for NAP1 expression and quantitative data of staining intensity were present in the left and right panels, respectively

To identify the clinical relevance of NAP1 in human cancer, we analyzed the expression levels of the *NAP1* gene among many types of cancer that were available in Oncomine. This analysis revealed that the majority of lung cancer patients had higher elevated expression levels of NAP1 in comparison to the other types of cancers analyzed (Fig. 1a). To study the clinical significance of NAP1 expression in lung cancer, we obtained the survival information of lung cancer patients from the Kaplan Meier-plotter. Univariate survival analysis revealed that high NAP1 expression levels were positively associated with low overall survival in patients with high-grade lung cancer (Fig. 1b). These results suggest that NAP1 may play a particularly important role in lung cancer. Moreover, a total of 65 NSCLC tissues with paired adjacent normal lung tissues were collected, and immunostained with anti-NAP1 antibody. Not surprisingly, NAP1 expression was significantly higher in tumor tissues than that in adjacent normal tissues (Fig. 1c). IHC data further showed that metastatic lung tumors expressed NAP1 exceptionally higher compared with non-metastatic lung tumors (Fig. 1d). A trend of increased NAP1 expression from noninvasive (G1), to superficially invasive (G2), and to deeply invasive (G3) lung tumor was also noted (Fig. 1e). This increasing trend was statistically significant at a P value less than 0.01 by ANOVA (Fig. 1e), supporting the involvement of NAP1 in the lethal and advanced forms of NSCLC.

### NAP1 upregulates MMP9 active form and promotes NSCLC cell invasion

**Fig. 2:**
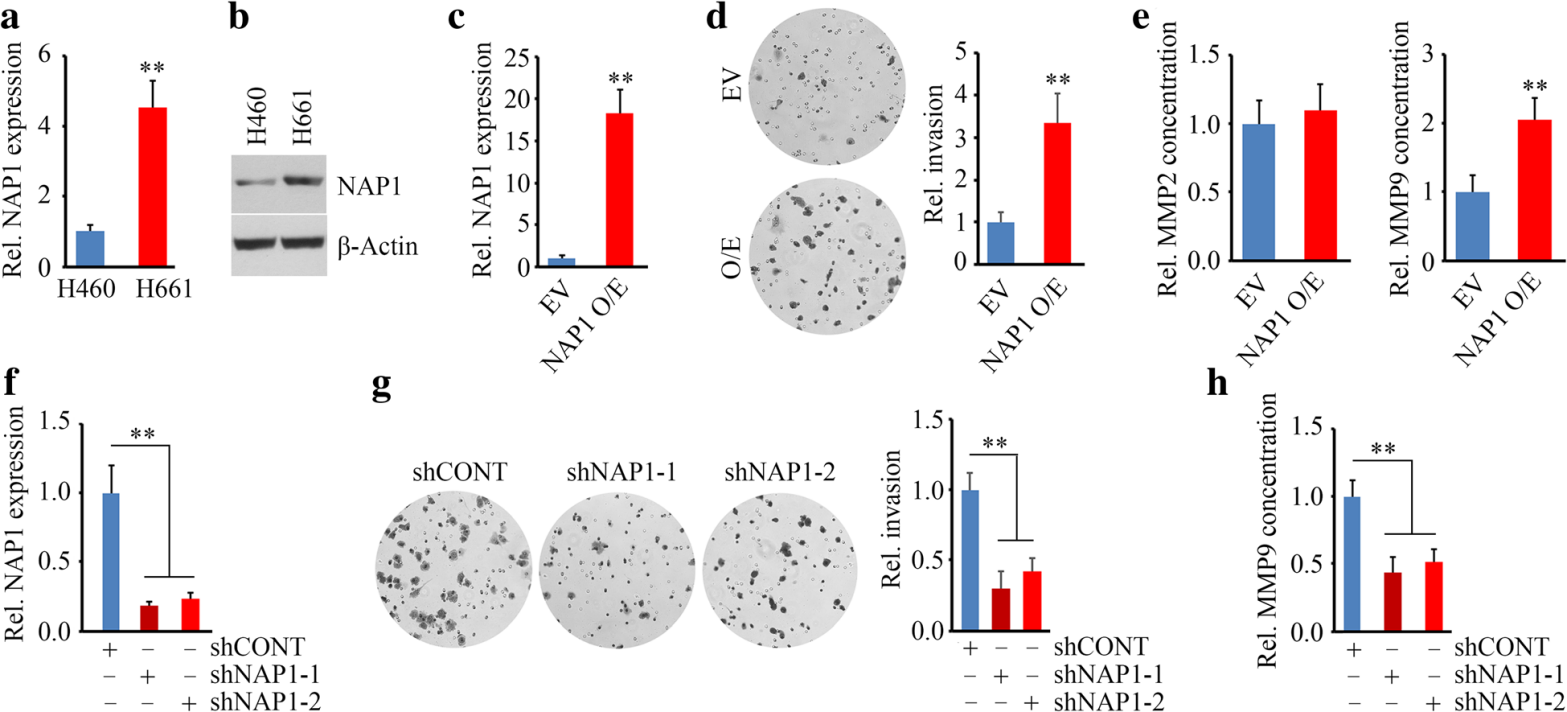
NAP1 promotes NSCLC cell invasion. (**a, b**) The expression levels of NAP1 in two NSCLC cell lines determined by qRT-PCR (**a**) and Western blotting (**b**). (**c**) Overexpression of NAP1 in H460 cells determined by qRT-PCR. (**d)** The effect of NAP1 overexpression on cell invasion determined by Transwell. (**e**) The effect of NAP1 overexpression on secretion of MMP2 and MMP9 in H460 cells determined by ELISA. (**f**) Knockdown of NAP1 in H661 cells determined by qRT-PCR. (**g**) The effect of NAP1 knockdown on cell invasion determined by Transwell. (**h**) The effect of NAP1 knockdown on MMP9 secretion in H661 cells determined by ELISA. EV: empty vector; NAP1 O/E: NAP1 overexpressing vector; shCONT: non-targeting shRNA; shNAP1–1 and shNAP1–2: two shRNAs against the *NAP1* gene. **p < 0.01

H460 is a low-invasive cell line derived from the pleural fluid of a patient with NSCLC, while H661 is a high-invasive cell line derived from the metastatic site of a patient with NSCLC. We first examined the expression levels of NAP1 in these two cell lines. As expected, NAP1 was expressed at higher levels in H661 cells compared with H460 cells (Fig. 2a and b), suggesting its potential importance in acquisition or maintenance of invasion. We then transfected H460 cells with full-length NAP1 (Fig. 2c) and determined the invasion in NAP1 overexpressing and control cells. Cells that overexpressed NAP1 exhibited much higher invasion potential compared with cells carrying an empty vector (Fig. 2d). No significant changes in proliferation were observed in cells with or without NAP1 knockdown (data not shown). Higher levels and activity of MMP2 and MMP9 have a significant association with the TNM stage of lung cancer and poor clinical evolution of patients with lung cancer [16]. Our previous studies revealed that NAP1 interacts with the WASF proteins that are associated with loss of MMP2 or MMP9 activity [5]. Therefore, we determined the activity of MMP2 and MMP9 in NSCLC cells when NAP1 expression was modified. ELISA analysis showed that the enzyme activity of MMP9, but not MMP2, was increased in supernatants from NAP1 overexpression H460 cells after 24 hours (Fig. 2e). In line with this finding, knockdown of NAP1 using shRNAs (Fig. 2f) significantly decreased invasion (Fig. 2g), and reduced the release of MMP9 from H661 cells (Fig. 2h).

**Fig. 3:**
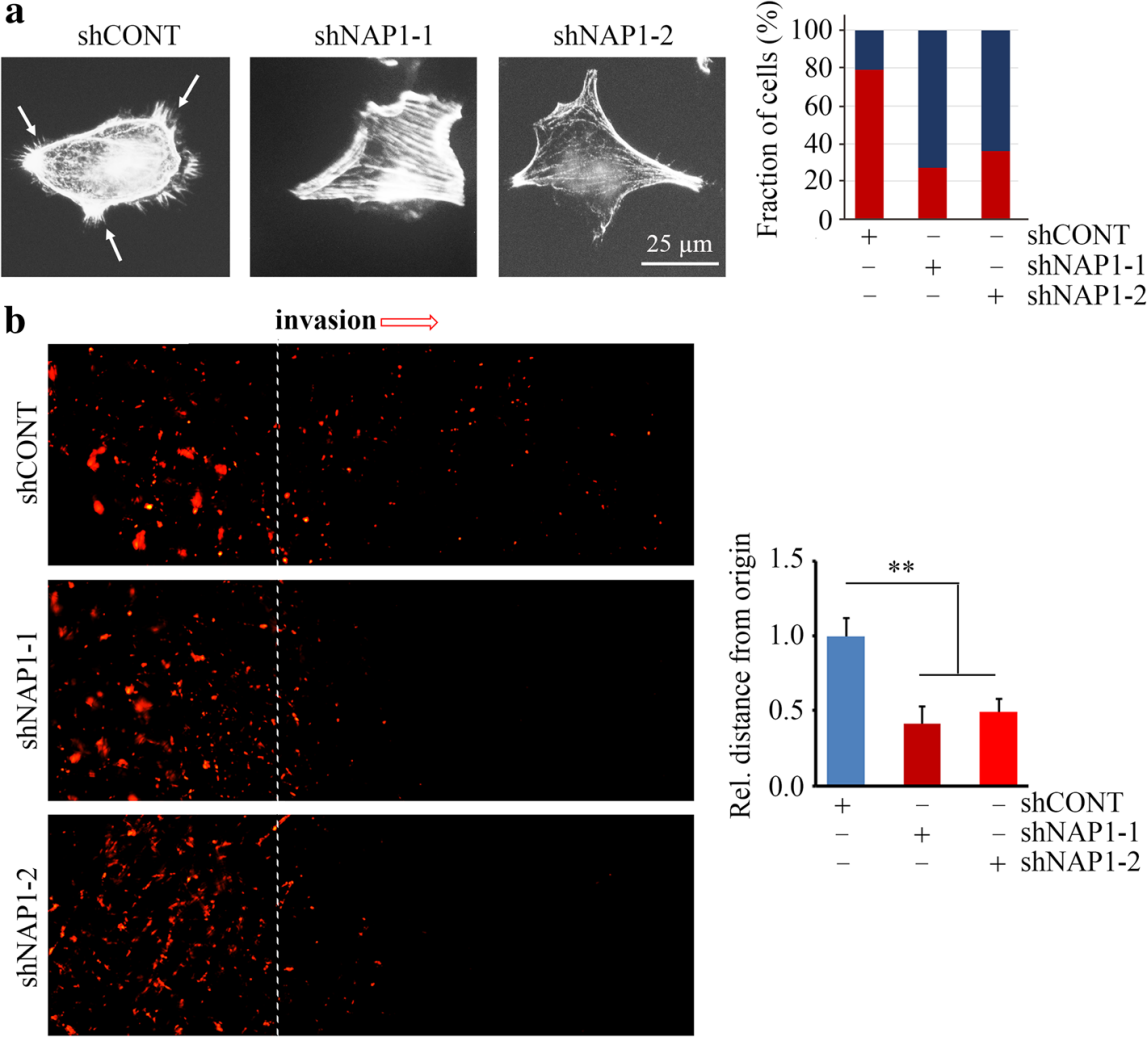
Loss of NAP1 expression suppresses H661 cell invasion in a 3D setting. (**a**) The effect of NAP1 knockdown on the number of lamelliapodia determined by phalloidin staining. (**b**) The effect of NAP1 knockdown on 3D in vitro invasion determined by SeedEZ™ rings. Representative images and quantitative data were present in the left and right panels, respectively

The capability of cells to form lamellipodia is most related to the migration and invasion potential. To determine whether the function of NAP1 is associated with the lamelipodia formation in NSCLC cells, H661 cells with or without NAP1 loss were stained with Texas-red phalloidin to reveal lamellipodial actin filaments. Depletion of NAP1 remarkably inhibited lamellipodia formation as evidenced by reduced number of lamellipodia protrusions in NAP1 knockdown cells (Fig. 3a). We next determined whether loss of NAP1 in NSCLC cells influenced the ability of H661 cell invasion in 3D scaffold using SeedEZ™, which showed the control cells invaded long distance while only a few cells with NAP1 loss displayed slight and short-distance invasion (Fig. 3b). Collectively, these observations support a notion that NAP1 is obligatory for lamellipodia formation and invasion of NSCLC cells.

### HSP90 enhances the stability of NAP1 protein in NSCLC cells

**Fig. 4:**
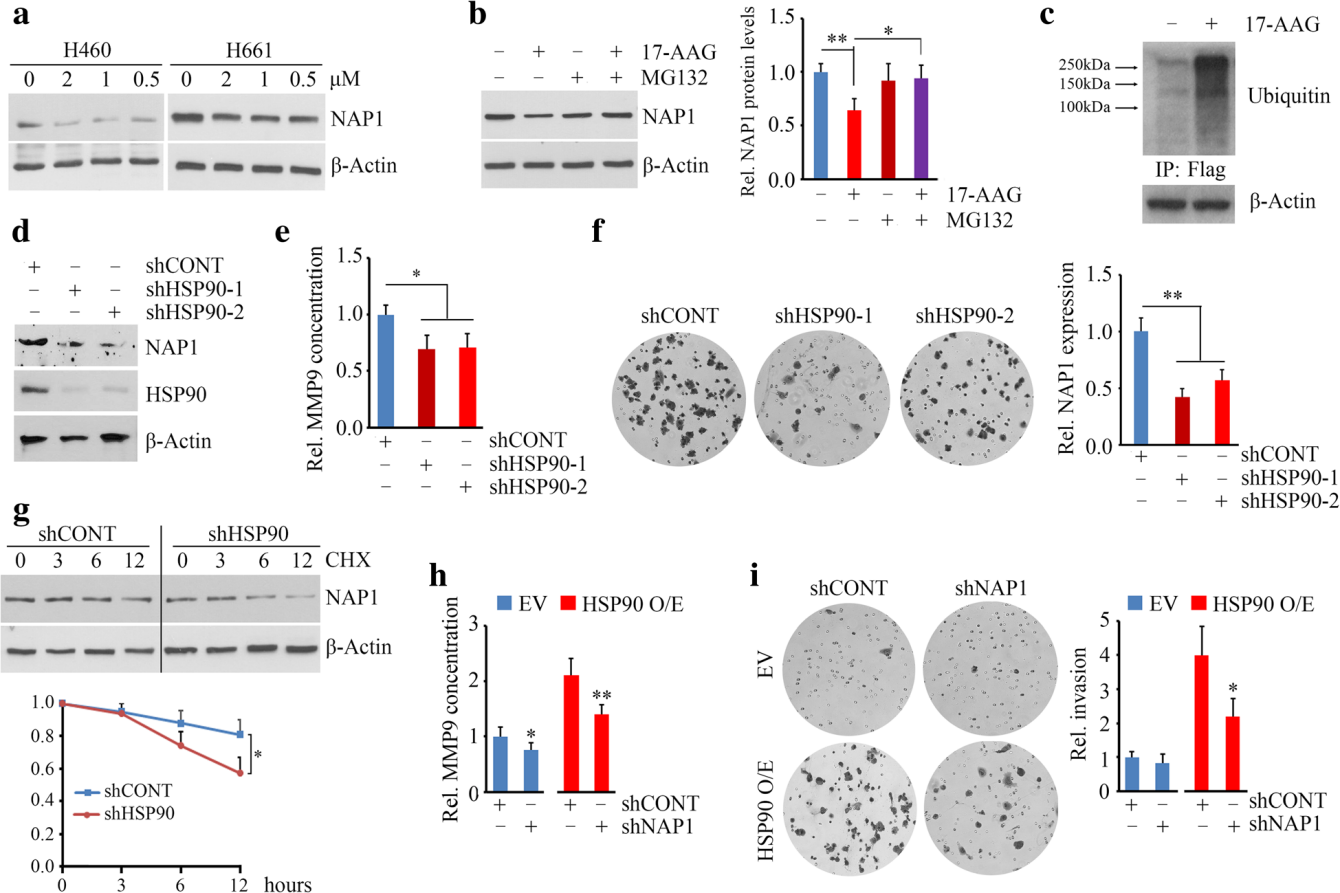
NAP1 is stabilized by HSP90 and contributes to HSP90-mediated invasion in NSCLC cells. **(a)** The effect of 17-AAG (0.5–2 μM) on NAP1 protein levels in two NSCLC cell lines. (**b**) The effect of 17-AAG on NAP1 protein levels in the presence or absence of MG132. H661 cells were pretreated with 10 μM MG132 for 4 h and then treated with 1 μM 17-AAG for 24 h. Representative Western blotting results and quantitative data were shown in the left and right panels, respectively. (**c**) The effect of 17-AAG on NAP1 ubiquitination in NAP1 overexpressing H460 cells. Cells were pretreated with 10 μM MG132 for 4 h before 17-AAG treatment. Cell lysates were IP using anti-Flag antibody and immunoblotted with anti-ubiquitin antibody. β-Actin immunoblotting on total lysate is shown to normalize the input. **(d)** The effect of HSP90 knockdown on NAP1 protein levels in H661 cells determined by Western blotting. (**e** and **f**) The effect of HSP90 knockdown on MMP9 secretion (**e**) and invasion (**f**) in H661 cells determined by ELISA and Transwell. (**g**) The effect of HSP90 knockdown on the half-life of NAP1 protein determined by CHX chase assays. HSP90 knockdown H661 cells were treated with 100 μg/ml CHX for the indicated hours. (**h** and **i**) The effect of NAP1 loss on MMP9 secretion (**h**) and invasion (**i**) in HSP90 overexpressing and control H661 cells. Representative results and quantitative data were present in the left and right panels, respectively. EV: empty vector; HSP90 O/E: the *HSP90* gene overexpressing vector; shCONT: non-targeting shRNA; shHSP90–1 and shHSP90–2: two shRNAs against the *HSP90* gene. *p < 0.05; **p < 0.01

HSP90 has been reported to promote invasion and activate MMP9 in lung cancer cells [17]. These phenotypes were also associated with NAP1 (Fig. 2), prompting us to determine a possible functional interaction between HSP90 and NAP1. HSP90 inhibitors have been developed for cancer treatment based on the fact that inhibiting HSP90-mediated protein folding machinery leads to simultaneous disruption of multiple oncogenic and metastatic pathways that are critical for the malignant phenotype of cancer cells. Among them, GA and 17-AAG function by binding to the HSP90 N-terminal ATP pocket, while novobiocin functions by binding to the HSP90 C-terminal nucleotide binding pocket [18]. These inhibitors were used to treat H661 cells for 24 hour followed by Western blotting with anti-NAP1 antibody. This analysis showed that either GA and 17-AAG or novobiocin have the ability to decrease NAP1 protein levels in H661 cells (Additional file 1: Figure S1), suggesting NAP1 may bind to the region of both C- and N-terminus of HSP90 in NSCLC cells. Intriguingly, at the same dose of 0.5 μM, 17-AAG exhibited a superior effect on NAP1 inhibition compared with GA and novobiocin (Additional file 1: Figure S1). Reduced NAP1 protein levels were also seen in H460 cells when 17-AAG was treated (Fig. 4a). Moreover, increasing the concentration of 17-AAG from 0.5 μM to 2 μM did not achieve better inhibitory effect on NAP1 in both H460 and H661 cells (Fig. 4a), indicating that 0.5 μM 17-AAG is sufficient to suppress NAP1. QRT-PCR further showed that 17-AAG-induced NAP1 repression was not a result of downregulation of NAP1 mRNA levels (data not shown). To determine whether NAP1 can be degraded through the proteasome upon 17-AAG exposure, HN661 cells were treated with the MG132 proteasome inhibitor followed by incubation of 17-AAG for 24 hours. As shown earlier, treatment with 17-AAG alone resulted in reduced NAP1 levels in H661 cells (Fig. 4b). MG132 treatment alone or in combination with 17-AAG showed no significant effect on NAP1 protein levels compared with non-treatment (Fig. 4b). To determine whether NAP1 undergoes polyubiquitination in 17-AAG treatment, MG132-pretreated NAP1 overexpressing H661 cells in the presence or absence of 17-AAG were immunoprecipitated with anti-flag antibody. The NAP1 immunocomplex displayed an increase in total ubiquitination in the presence of 17-AAG (Fig. 4c).

To better define the specific role of HSP90 in the stabilization of NAP1 protein, we depleted HSP90 expression in H661 cells using lentiviral shRNA constructs. Decreased NAP1 protein levels were observed when HSP90 was knocked down (Fig. 4d). Moreover, HSP90 knockdown cells reduced active MMP9 levels (Fig. 4e) and their invasion abilities (Fig. 4f), which were similar to the observations from NAP1 knockdown cells. We next determined the importance of HSP90 in NAP1 function in NSCLC cells. H661 cells were treated with CHX to block protein synthesis. This treatment showed a limited effect on knockdown control cells; however, it led to a remarkable reduction in NAP1 protein levels as a result of HSP90 knockdown (Fig. 4g). Moreover, overexpression of HSP90 enhanced the accumulation of active MMP9 in cell supernatant and invasion of H661 cells, but this increase was attenuated when NAP1 was depleted (Fig. 4h and i). Taken together, these findings demonstrate that the functional interaction between HSP90 and NAP1 proteins plays a critical role in NSCLC cell invasion.

### Loss of HSP90 suppresses NAP1-mediated NSCLC metastasis in mice

**Fig. 5:**
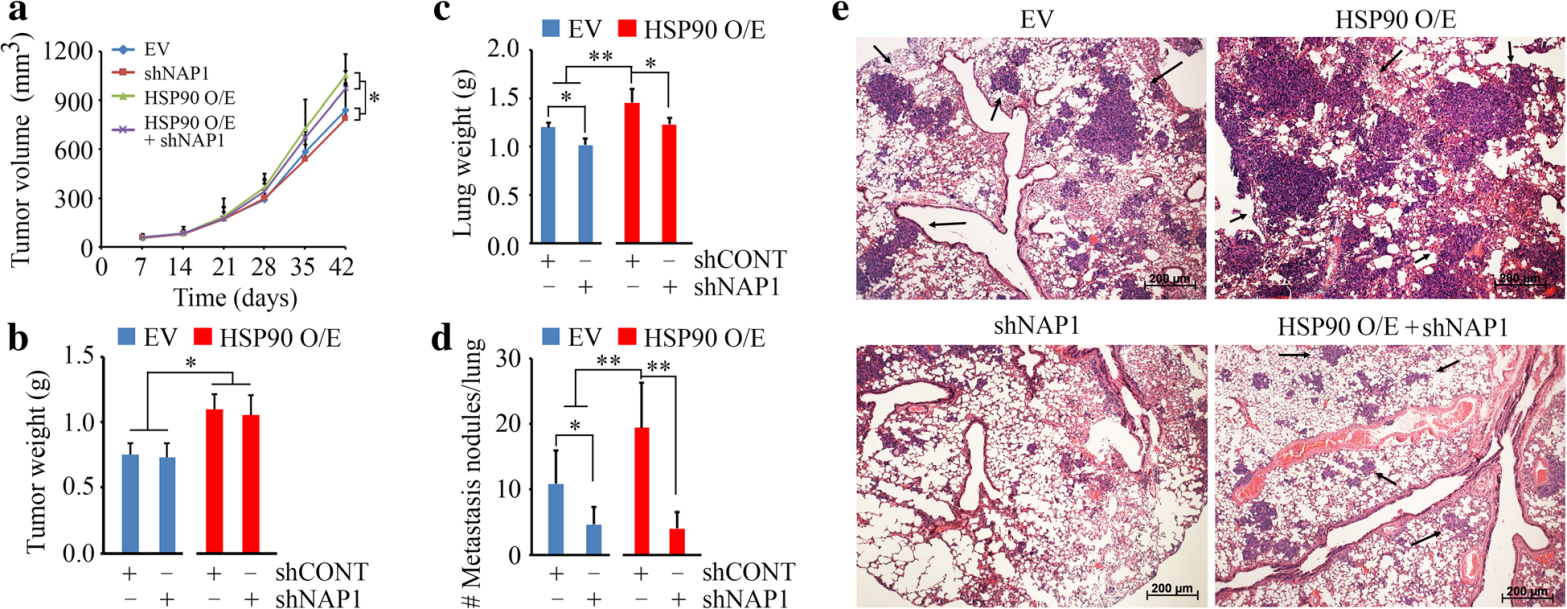
Loss of NAP1 suppresses HSP90-mediated metastasis of NSCLC cells in mice. (**a**) Tumor growth measured by tumor volume within 42 days. (**b**-**d**) Tumor (**b**) and lung (**c**) weight and the number of pulmonary nodules (**d**) measured on day 42. (**e**) Lung micro-metastasis determined by H&E staining. The six-week old NSG mice were randomly assigned to four groups (*n* = 5) to receive H661 cells carrying the indicated gene modifications. EV: empty vector; HSP90 O/E: the *HSP90* gene overexpressing vector; shNAP1: shRNA against the *NAP1* gene. *p < 0.05; **p < 0.01

Metastasis is the complex process by which primary tumor cells metastasize and establish secondary tumors in an adjacent or distant location in the body. In line with the *in vitro* findings, HSP90 overexpressing H661 cells with or without NAP1 knockdown and their control cells were subcutaneously inoculated into NSG mice. After 42-day inoculation, larger tumors were seen in mice receiving HSP90 overexpressing cells compared with the control groups (Fig. 5a and b). Interestingly, no significant changes in tumor growth were observed in mice receiving NAP1 knockdown or control cells, with similar tumor size and weight (Fig. 5a and b). Notably, less NAP1 knockdown cells established colonies on the surface of lung compared with control cells (Fig. 5c and d). In contrast, overexpression of HSP90 remarkably enhanced metastatic potential of H661 cells to the lung (Fig. 5c and d). Consistent with the *in vitro* invasion data, knockdown of NAP1 significantly attenuated metastatic potential induced by HSP90 overexpression as evidenced by reduced nodule numbers on the lung surface (Fig. 5c and d) and decreased infiltrating tumors throughout the entire lung (Fig. 5e). These results demonstrate that NAP1 promotes metastasis of NSCLC relying on HSP90 function for protein stability.

### NAP1 is a novel HSP90-interacting protein and required for its mediated Vimentin in NSCLC cells

**Fig. 6:**
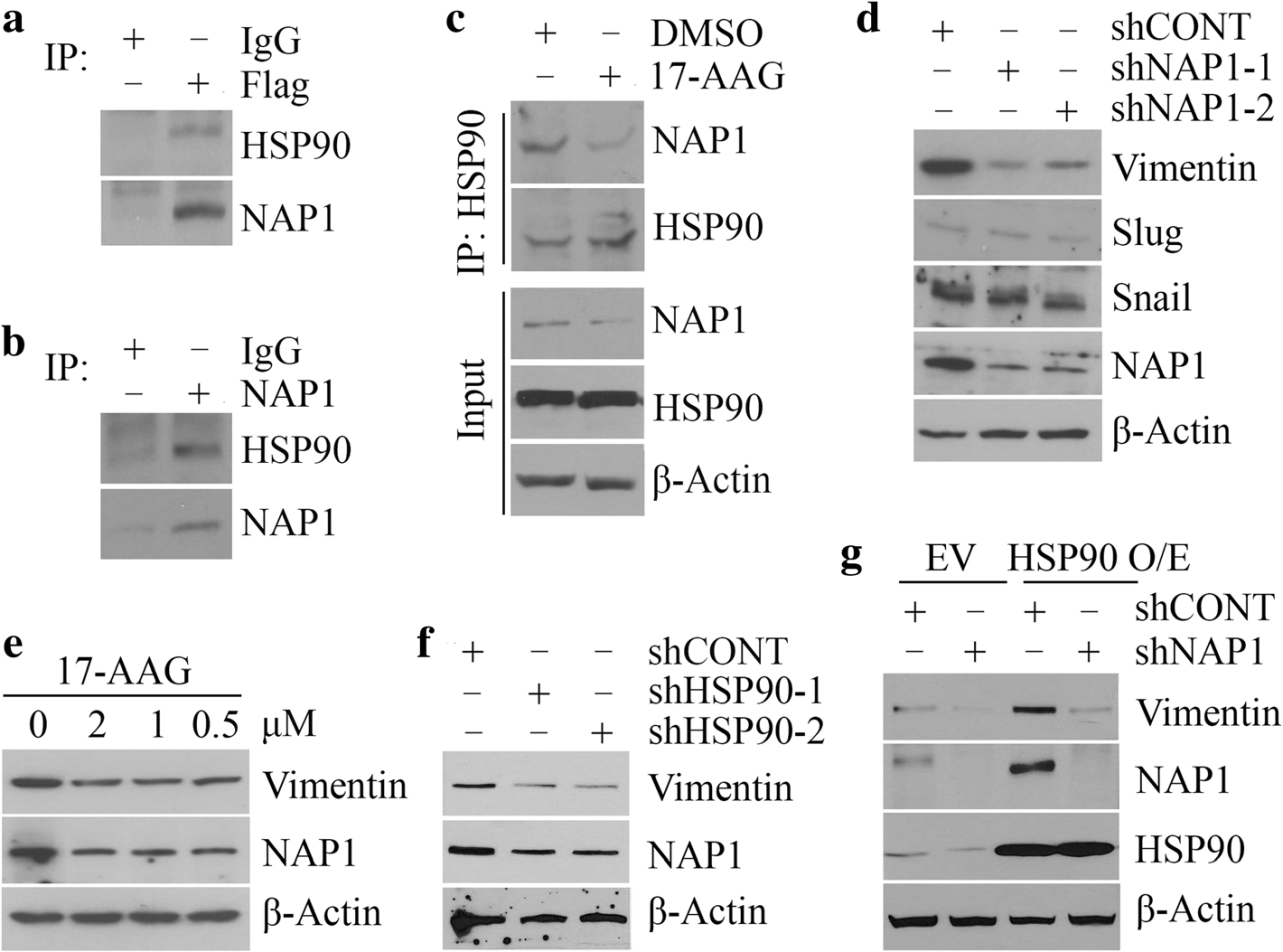
NAP1 is a new HSP90-interacting partner and required for its mediated Vimentin in NSCLC cells. (**a** and **b**) The interaction between HSP90 and NAP1 proteins determined by IP. The immunoprecipitates from NAP1 overexpressing H460 cells (**a**) and H661 cells (**b**) were pulled down with anti-Flag and anti-NAP1 antibodies, respectively, followed by Western blotting with antibodies against HSP90 and NAP1. Preimmune IgG was used as a control. (**c**) The effect of 17-AAG on the HSP90-NAP1 interaction. (**d**) The effect of NAP1 knockdown on molecules related to EMT. (**e** and **f**) The effect of 17-AAG (**e**) and HSP90 knockdown (**f**) on Vimentin protein levels. (**g**) The effect of NAP1 knockdown on Vimentin protein levels in HSP90 overexpressing and control cells. In (**c-g**), H661 cells were used as a cell model. EV: empty vector; HSP90 O/E: the *HSP90* gene overexpressing vector

To determine whether NAP is one of HSP90-interacting partners, we immunoprecipitated NAP1 protein with anti-Flag antibody in NAP1 overexpressing H460 cells, which identified HSP90 in the immunocomplex (Fig. 6a). The interaction between HSP90 and NAP1 was also demonstrated in H661 cells with high levels of endogenous NAP1 (Fig. 6b), suggesting a wider association between these two proteins. We then immunoprecipitated HSP90 immunocomplex from H661 cells in the presence or absence of 17-AAG, which showed that 17-AAG not only reduced NAP1 protein levels but also blocked its association with HSP90 (Fig. 6c). These observations indicate that HSP90 promotes NAP1 protein stability through interacting with NAP1.

As the epithelial-to-mesenchymal transition (EMT) is a key driver of cancer metastasis [5, 12], we determined the changes in several EMT-related proteins in NAP1 knockdown and control H661 cells, which showed dramatically reduced levels of Vimentin protein along with NAP1 depletion (Fig. 6d). We next determined whether loss of HSP90 influenced Vimentin protein. Either 17-AAG treatment or shRNA-mediated HSP90 knockdown led to a decrease in Vimentin protein levels, compared with the corresponding controls (Fig. 6e and f). Overexpression of HSP90 in H661 cells produced opposing effect, resulting in increased Vimentin levels (Fig. 6g). Most importantly, knockdown of NAP1 markedly abrogated the amount of Vimentin induced by HSP90 overexpression (Fig. 6g), suggesting that NAP1 plays an essential role in the regulation of Vimentin by HSP90 in NSCLC cells.

## Discussion

Alterations of the cytoskeletal protein actin, a major structure and motility factor in the cell, are critical for cancer invasion and metastasis. However, how actin is associated with malignant phenotypic changes and other exact mechanisms remain to be fully elucidated. We report here that NAP1, an important regulator of actin cytoskeleton dynamics, plays an essential role in driving NSCLC progression towards metastasis without affecting tumorigenesis and tumor growth. The clinical relevance and significance of NAP1 were evidenced by the positive correlation between high expression levels of NAP1 and metastasis and the poor prognosis of lung cancer patients. Mechanistically, HSP90 interacts with NAP1 to prevent it from ubiquitin-proteasome-dependent degradation, and the stabilization of the HSP90-NAP1 axis appears to promote invasion and metastasis of NSCLC. Thus, the present study provides a rational basis for interruption of the HSP90-NAP1 protein complex in NSCLC cells as a potential anti-metastatic therapeutic.

Previous studies have suggested a high expression of NAP1 in the human brain [19]. In our IHC study, no or very low expression levels of NAP1 were determined in human lung tissues, suggesting a tissue-specific expression pattern of NAP1. Nevertheless, NAP1 is highly expressed in NSCLC, which is associated with tumor grade and metastasis potential. This novel finding is different from the results of other investigations of NAP1 in hepatocellular carcinoma (HCC), which showed that NAP1 was negatively regulated by miR-34c-3p and its low expression was associated with a favorable prognosis of HCC patients [20]. A similar NAP1 expression pattern has been seen in breast cancer, and knockdown of NAP1 in this type of cancer remarkably suppresses tumor metastasis in a highly preclinical animal model [5]. In view of the positive association between increased NAP1 expression levels and tumor progression identified in these studies, caution should be taken in the effort of developing therapeutic interventions based on NAP1 regulatory strategies for both breast and lung cancer.

HSP90, also termed the ‘cancer chaperone’, is itself often overexpressed in cancer cells and regarded as essential for maintaining the stability and activity of numerous signaling proteins involved in metastasis processes [17]. For instance, inactivation of HSP90 by GA stimulates AKT and ERK via transient activation of Src resulting from dissociation of Src from HSP90 [21]. Our study provides the first evidence that NAP1 is ‘clients’ of HSP90, in that it relies on HSP90 for proper folding, stability and function. HSP90 exists not only in the intracellular space, but also in the extracellular space. Extracellular HSP90 seems to regulate cell motility through matrix-degrading proteases and cell surface receptors. However, few studies have been reported as to how intracellular HSP90 contributes to driving cancer progression towards metastasis. Our novel observations that HSP90 binds to and stabilizes NAP1 in NSCLC cells may help clarify the functions of intracellular HSP90. HSP90 inhibitors have been shown to display promising therapeutic activity for cancer treatment; however, they also have adverse effects on normal cells [22]. Therefore, selective disassociation of NAP1 from HSP90 by new drugs provides a possible strategy to dampen the metastatic signal from the HSP90-NAP1 protein complex.

The human HSP90 family encompasses 5 gene products differing from each other by expression level, subcellular location and amino acid constitution [23–25]. The two major cytosolic HSP90s cover the inducible HSP90α and the constitutive HSP90β, which are associated with cancer cell survival, growth and metastasis [23–25]. Other three isoforms, including GRP94, HSPC4 and TRAP1, can be found in the endoplasmic reticulum (ER), mitochondria, and the nucleus [23–25]. In the present study, we employed the anti-HSP90α monoclonal antibody and HSP90α overexpression plasmid to determine and modulate HSP90 levels; therefore, the results from this study can only indicate the functional regulation of NAP1 by HSP90α. Given that upregulation of other HSP90 isoforms, including the cytosolic HSP90β, ER resident GRP94 and mitochondrial localized TRAP1, has been reported in NCSCL [26–29], vigorous research efforts are needed to further clarify whether their functions associated with cancer development and progression are also through the NAP1 signaling.

The stability of WASF proteins (WASF1, 2 and 3) are regulated by NAP1 within the WRC [5]. WASF proteins have been shown to regulate cancer invasion and metastasis by cytoskeletal actin-associated motility machinery [30]. Among them, WASF3 is a well-characterized protein controlling breast cancer metastasis [5, 6, 18, 31]. Our previous study has shown that knockdown of WASF3 upregulates KISS1, a metastasis suppressor, with concomitant reduced invasion and MMP9 activity in breast cancer cells [32]. As shown in this study, NAP1 functions to activate MMP9 in NSCLC cells, which profoundly advances the understanding of mechanisms of action in cancer metastasis. Further investigation is warranted to delineate how the WASF3-KISS-MMP9 signaling axis contributes to NAP1-driven NSCLC metastasis.

## Conclusion

Interaction of NAP1 to HSP90 attenuates proteasome-dependent degradation of NAP1, leading to NSCLC metastasis by provoking the activation of MMP9 and EMT. The present work provides novel insight into how the HSP90-NAP1 signaling axis regulates metastasis and generates a strong mechanism-based framework for developing therapeutics that block NSCLC metastasis.

## Additional file

Additional file 1: **Figure S1.** 17-AAG exhibits a superior effect on suppression of NAP1 proteins compared with other two HSP90 inhibitors. (**a, b**) H661 cells were treated with 0.5 μM different HSP90 inhibitors: geldanamycin (GA), novobiocin (Novo) or 17-AAG, for 24 h, and the protein levels of NAP1 was determined by Western blotting. Representative and quantitative data were shown in (**a**) and (**b**), respectively. *p < 0.05; **p < 0.01. (TIF 185 kb)

## Abbreviations

17-AAG: 17-allylamino-demethoxygeldamycin
CHX: Cycloheximide
ER: Endoplasmic reticulum
GA: Geldanamycin
HSP90: Heat shock protein 90
IHC: Immunohistochemistry
IOD: Integrated optical density
MMP2: Matrix metalloproteinase 2
MMP9: Matrix metalloproteinase 9
NCKAP1/NAP: Nck-associated protein 1
Novo: Novobiocin
NSCLC: Non-small-cell lung cancer
NSG: NOD.Cg*-Prkdc*^*scid*^ *Il2rg*^*tm1Wjl*^*/SzJ*
SH3: Src homology 3
shRNA: short hairpin RNA
WRC: WASF regulatory complex
