# Supplementary figures and images for: Nck-associated protein 1 associates with HSP90 to drive metastasis in human non-small-cell lung cancer
Source: J Exp Clin Cancer Res. 2019 Mar 11;38:122. doi: 10.1186/s13046-019-1124-0 (PMC6417146; doi:10.1186/s13046-019-1124-0)

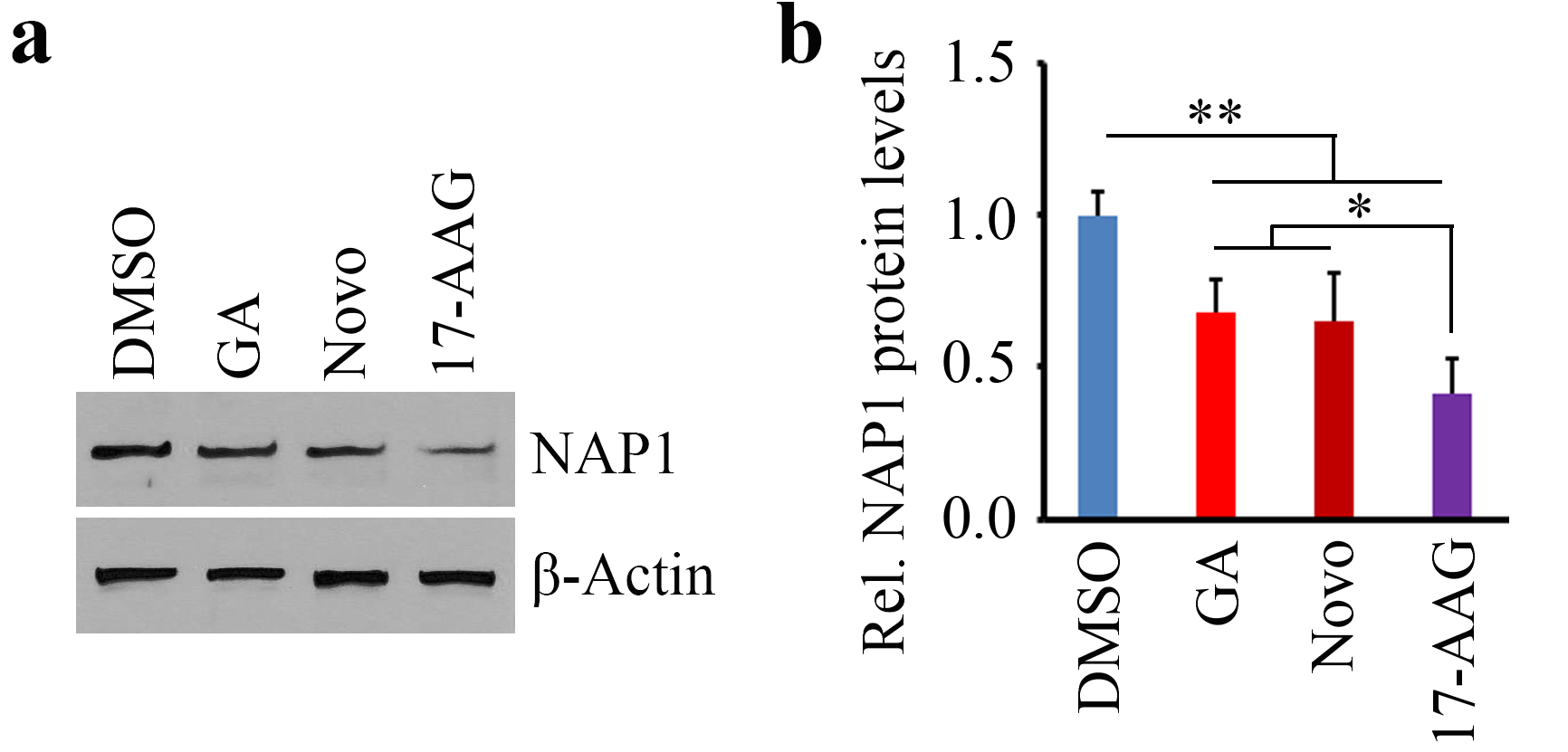

Supplement: Supplementary file 1 — Figure S1. 17-AAG exhibits a superior effect on suppression of NAP1 proteins compared with other two HSP90 inhibitors. (a, b) H661 cells were treated with 0.5 μM different HSP90 inhibitors: geldanamycin (GA), novobiocin (Novo) or 17-AAG, for 24 h, and the protein levels of NAP1 was determined by Western blotting. Representative and quantitative data were shown in (a) and (b), respectively. *p < 0.05; **p < 0.01. (TIF 185 kb) [file 13046_2019_1124_MOESM1_ESM.tif]
